# Knowledge Mapping of Olfactory Dysfunction: A Bibliometric Study

**DOI:** 10.3389/fnsys.2022.904982

**Published:** 2022-06-13

**Authors:** Wen Hu, Na Chen, Weiheng Yan, Pei Pei, Yongxiang Wei, Xiaojun Zhan

**Affiliations:** ^1^Department of Otolaryngology, Smell and Taste Center, Beijing Anzhen Hospital, Capital Medical University, Beijing, China; ^2^Department of Otolaryngology, Beijing Rehabilitation Hospital, Capital Medical University, Beijing, China; ^3^Department of Otorhinolaryngology Head and Neck Surgery, Children's Hospital, Capital Institute of Pediatrics, Beijing, China; ^4^Graduate School of Peking Union Medical College, Chinese Academy of Medical Sciences, Beijing, China; ^5^Beijing Municipal Key Laboratory of Child Development and Nutriomics, Capital Institute of Pediatrics, Beijing, China

**Keywords:** bibliometric study, CiteSpace, olfactory dysfunction, VOSviewer, hotspot, trend

## Abstract

**Background:**

Olfaction is one of the five basic senses of human beings. As such, olfactory dysfunction seriously affects patients' quality of life and can even endanger them. In recent years, olfactory dysfunction has attracted greater research interest, and numerous studies have been published on olfactory dysfunction. However, there are few studies on olfactory dysfunction through bibliometric analysis. This study aims to describe the current situation and identify the foci and potential new research directions of olfactory dysfunction using a bibliometric approach.

**Methods:**

Articles related to olfactory dysfunction published from 2002 to 2021 were located in the Web of Science Core Collection of Clarivate Analytics (London, UK). Bibliometric analyses were conducted with the CiteSpace (Chaomei Chen, Drexel University, Philadelphia, PA, USA) and VOSviewer (Center for Science and Technology Studies, Leiden University, Leiden, Netherlands) software programs.

**Results:**

The number of articles published each year showed an upward trend, especially in 2020, where a sharp increase had occurred due to the coronavirus disease 2019 (COVID-19) pandemic. The United States was the country with the most publications and the strongest international cooperation. In terms of institutions, the greatest number of publications from a single institution came from Dresden University of Technology. Thomas Hummel was the author who had contributed the most articles. An analysis of co-citation networks and burst keywords in the field revealed a shift from “gonadotropin-releasing hormone” and “apoptosis” earlier on to “olfactory training,” “COVID-19,” and “Parkinson's disease” more recently. “Outcome,” “COVID-19,” “infection,” and “pathogenesis” are topics of the research frontier and hotspots.

**Conclusion:**

More attention has been paid to olfactory dysfunction as the understanding of it has improved in the past 20 years. This study provides researchers with an objective, systematic, and comprehensive analysis of the literature on olfactory dysfunction. The current frontier areas and hotspots in the field focus on the pathological mechanisms of olfactory dysfunction after infection with COVID-19 and its different prognoses. The pathophysiological mechanism of olfactory dysfunction in neurodegenerative diseases and COVID-19 will be a primary future research direction.

## Introduction

The olfaction plays an extremely important role in human behavior, social interaction, and daily life. Apart from affecting mental activities in terms of learning, memory, and arousal, it is associated with the normal development of sensation, cognition, mood, and emotion in childhood (Pinto et al., 2015; Meissner-Bernard et al., 2019). As such, olfactory dysfunction not only affects quality of life, social interaction, nutrient intake, and life safety but may also lead to emotional disorders such as depression and psychiatric disorders (Vennemann et al., 2008). Olfactory dysfunction was previously thought to affect ≥1% of the population, but this figure has now reached >20% (Landis et al., 2004; Nordin and Brämerson, 2008; Vennemann et al., 2008) according to the latest epidemiological surveys. Furthermore, olfactory dysfunction is an early warning indicator of some severe diseases. Olfactory dysfunction is a common early symptom in neurodegenerative diseases such as Alzheimer's disease and Parkinson's disease (Domellöf et al., 2017). A reduction in olfactory function even enhances the risk of mortality within 5 years (Pinto et al., 2014). Olfactory dysfunction is also an important early sign by which to diagnose major respiratory infectious diseases. In patients with severe acute respiratory syndrome coronavirus 2 (SARS-CoV-2) infection, the incidence of olfactory dysfunction ranges from 5.1 to 83%, and olfactory dysfunction is the only early symptom experienced by some patients (Ibekwe et al., 2020). According to several studies, upper respiratory tract infections, inflammation, and trauma are the most common clinical causes of olfactory dysfunction, accounting for 36, 30, and 18% of cases, respectively (Deems et al., 1991; Chen et al., 2013).

Olfactory dysfunction is usually considered a disorder that is difficult to treat. Currently employed treatments for olfactory dysfunction include drug therapy, surgery, olfactory training, and other methods. Drug therapy generally involves glucocorticoids, with an effective rate of 25–50% (Heilmann et al., 2004). Olfactory training is an effective method to treat olfactory dysfunction after upper respiratory tract infection (Hummel et al., 2009). However, the overall effectiveness of these treatments is low (Hura et al., 2020). During the past two decades, researchers around the world have made great advances in the epidemiology, pathophysiology, diagnosis, and treatment of olfactory dysfunction. Understanding the current hotspots and frontiers in the research field of olfactory dysfunction is helpful for researchers seeking to conduct relevant in-depth research.

With the advent of the era of big data, scientific innovation based on scientific data mining has become an important tool. Through quantitative analysis of a large number of papers in a certain field, bibliometric analysis estimates the contribution of that field, reveals research hotspots, and predicts the future direction of the selected research field (Cebral-Loureda et al., 2022; Lin et al., 2022). A scientometric analysis of the papers is mainly performed using CiteSpace (Chaomei Chen, Drexel University, Philadelphia, PA, USA) and VOSviewer (Center for Science and Technology Studies, Leiden University, Leiden, Netherlands). CiteSpace is a JAVA-based mapping software that enables metrics, co-occurrence analysis, cluster analysis, and data visualization (Chen, 2004; Synnestvedt et al., 2005). VOSviewer is a software for constructing text maps based on web data.

To date, few scientometric studies on olfactory dysfunction have been reported (Zyoud et al., 2022). The present study evaluates the literature on olfactory dysfunction published from 2002 to 2021 to describe the current state of this field and predict potential development trends in the next few years.

## Materials and Methods

### Data Collection

**Figure 1:**
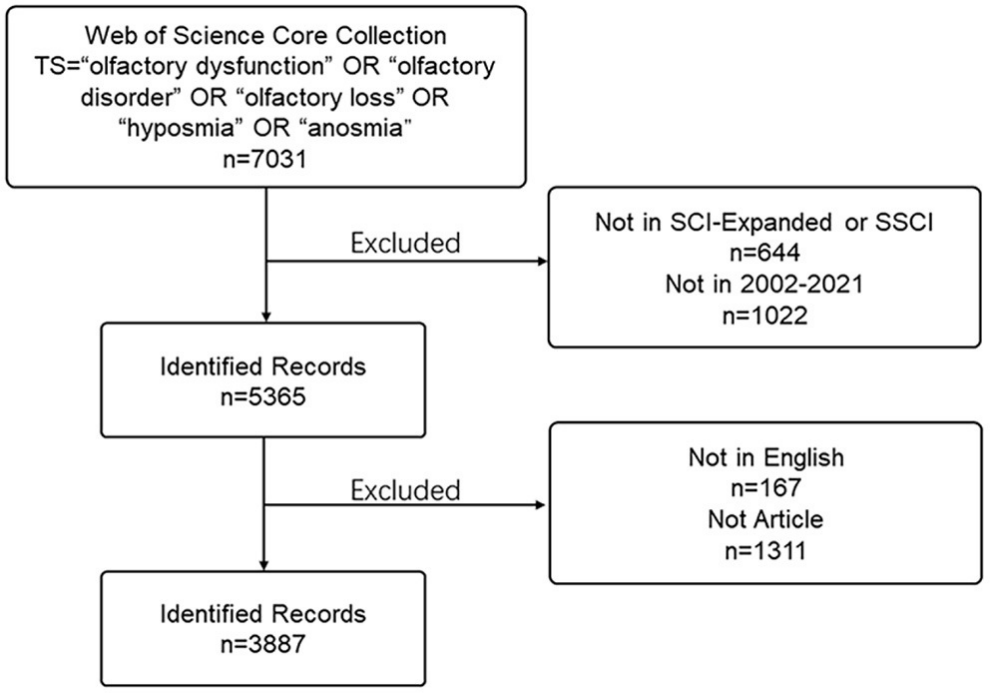
Flow diagram of the literature search.

Literature related to olfactory dysfunction were searched for on March 3, 2022, using the Science Citation Index—Expanded and the Social Sciences Citation Index of the Web of Science Core Collection (WoSCC). The main search terms were “olfactory dysfunction,” “olfactory disorder,” “olfactory loss,” “hyposmia,” and “anosmia.” Original articles published in English from 2002 to 2021 were retrieved. The detailed search procedure is presented in Figure 1. Two authors searched the WoSCC database independently and downloaded information on titles, abstracts, keywords, authors, institutions, countries, journals, references, and citations in.txt format. Divergent viewpoints were settled by discussion or by seeking the help of a third party.

### Analysis Method

The searched studies were downloaded and imported into CiteSpace 5.8.R4, 64-bit; VOSviewer 1.6.16; and the bibliometric online analysis platform (http://bibliometric.com/) to perform a visualization analysis. CiteSpace was used to analyze the details of the identified articles, including publication countries/regions and institution, authors, journals, co-cited references, and burst keywords. The parameters of CiteSpace were as follows: time span (2002–2021), year of slice (1 year), selection criteria (top 50), and visualization (cluster view-static and show merged network). VOSviewer 1.6.16 was used to construct scientifically based knowledge networks, including publication countries/regions, journals, and keywords. Microsoft Excel 2016 (Microsoft Corporation, Redmond, WA, USA) was used to construct a polynomial regression model to predict the number of articles related to olfactory dysfunction published in 2022. Ethical approval was not necessary in this study.

## Results

### Annual Publications

**Figure 2:**
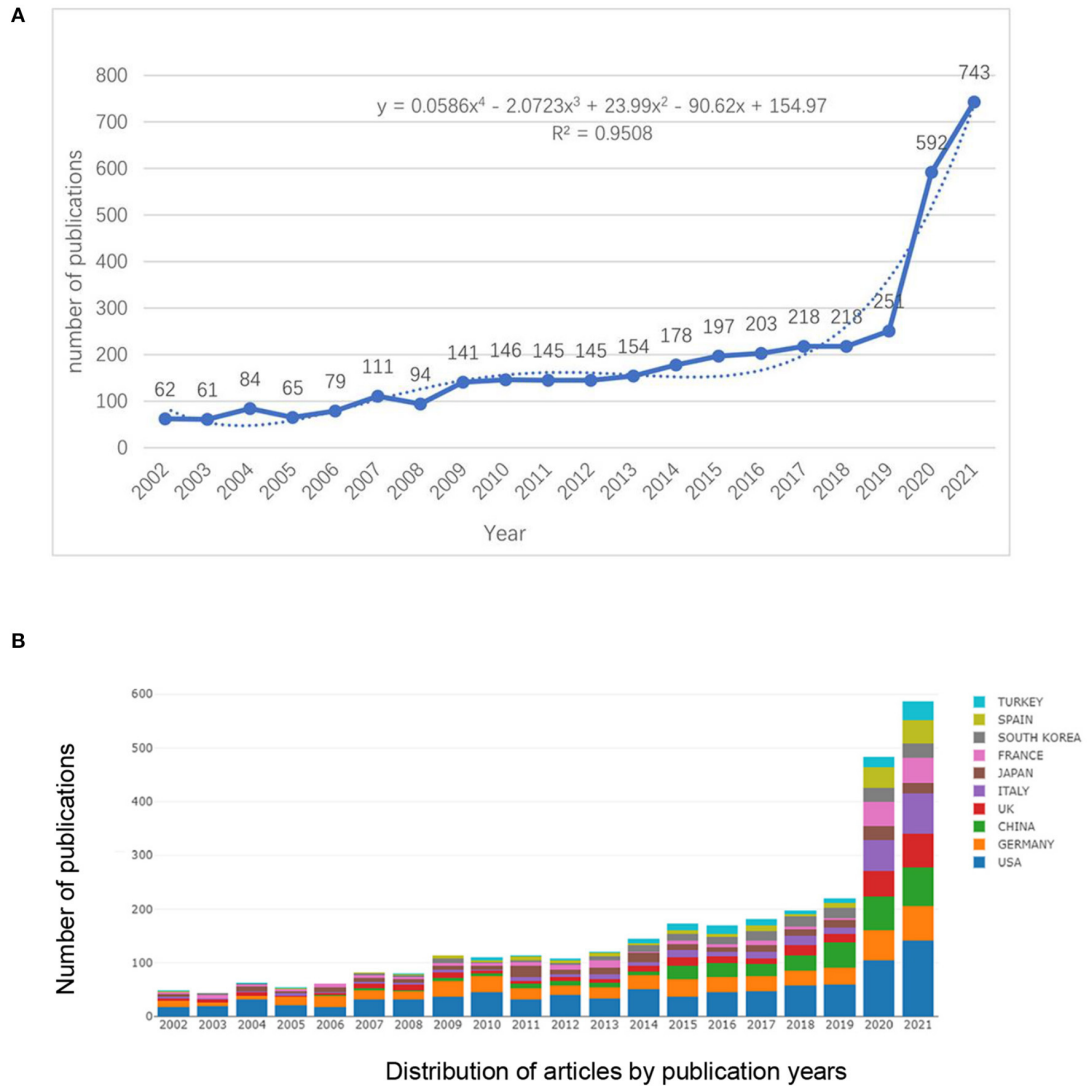
The number and polynomial curve fitting of publications **(A)** and top 10 countries or regions publishing olfactory dysfunction research between 2002 and 2020 **(B)**.

A total of 3,887 articles about olfactory dysfunction published between 2002 and 2021 were retrieved after searching the WoSSC database. The olfactory dysfunction research output exhibited a growth trend (Figure 2A). Over the past 20 years, the United States (U.S.) has maintained the highest annual publication rate of original articles (Figure 2B). From 2003 to 2019, the number of publications maintained a steady growth rate. Then, from 2020 onward, the number of publications increased rapidly; more than double the number of papers published in 2019 were published in 2020.

By fitting the data, a statistically significant relationship could be observed between publication year and the number of publications (*R*^2^ = 0.9508). Based on the polynomial fitted curve, we estimated that the number of original articles related to olfactory dysfunction will reach 1,036 in 2022.

### Distribution of Countries/Regions and Institution

**Figure 3:**
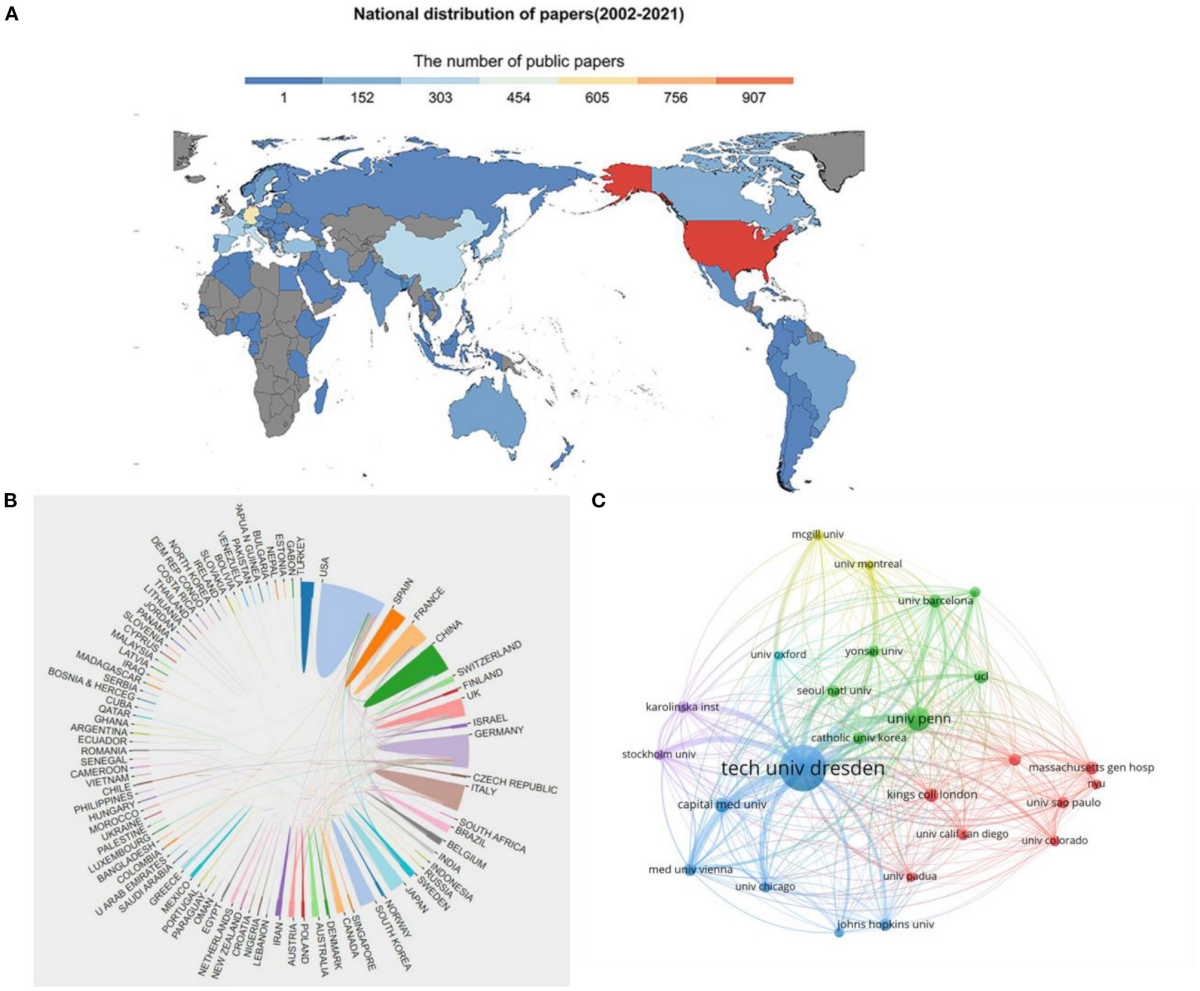
The distribution of countries/regions in terms of publications **(A)** and the co-operation of countries/regions **(B)**. A network map showing the institutions involved in research of olfactory dysfunction **(C)**.

The retrieved articles in this study hailed from 100 countries. As shown in the country distribution chart (Figure 3A), the countries with the highest number of published papers were the U.S., Germany, and China, respectively. In contrast, few countries in Africa have contributed to the literature. In CiteSpace, Centrality is used to quantify the importance of a node's position in the network. The centrality analysis showed that the U.S. (0.36) was the core of the network, followed by the United Kingdom (U.K.) (0.17) and Italy (0.12) (Table 1). Figure 3B displays the network of national collaborations. The U.S., Germany, the U.K., and Italy are the top countries that have maintained intensive cooperation with other countries/regions.

**Table 1:** Top 10 most published countries in the field of olfactory dysfunction from 2002 to 2021.

**Rank**	**Country**	**Count**	**Centrality**
1	USA	1,020	0.36
2	Germany	472	0.07
3	China	349	0.04
4	Italy	308	0.12
5	UK	300	0.17
6	France	205	0.07
7	Japan	191	0.02
8	South Korea	180	0.07
9	Turkey	179	0.04
10	Spain	175	0.04

Figure 3C illustrates the cooperation network of institutions. Dresden University of Technology (tech univ dresden) (*n* = 285), the University of Pennsylvania (univ penn) (*n* = 105), and the University of London (ucl) (*n* = 95) ranked as the top three institutions in terms of the number of published articles (Table 2); these institutions were also the top three institutions in terms of centrality. The centrality of these three institutions was >0.1, indicating that they have a certain influence in the research field of olfactory dysfunction.

**Table 2:** Top 10 institutions with the most publications in the field of olfactory dysfunction from 2002 to 2021.

**No**.	**Institution**	**Country**	**Count**	**Centrality**
1	Dresden University of Technology	Germany	285	0.31
2	University of Pennsylvania	USA	105	0.11
3	University of London	UK	95	0.03
4	Capital Medical University	China	50	0.03
5	Massachusetts General Hospital	USA	42	0.03
6	University of Barcelona	Spain	42	0.06
7	Kings College London	UK	42	0.1
8	Karolinska Institutet	Sweden	41	0.04
9	Harvard Medical School	USA	41	0.01
10	Medical University of Vienna	Austria	38	0.01

### Contributions of Authors

**Figure 4:**
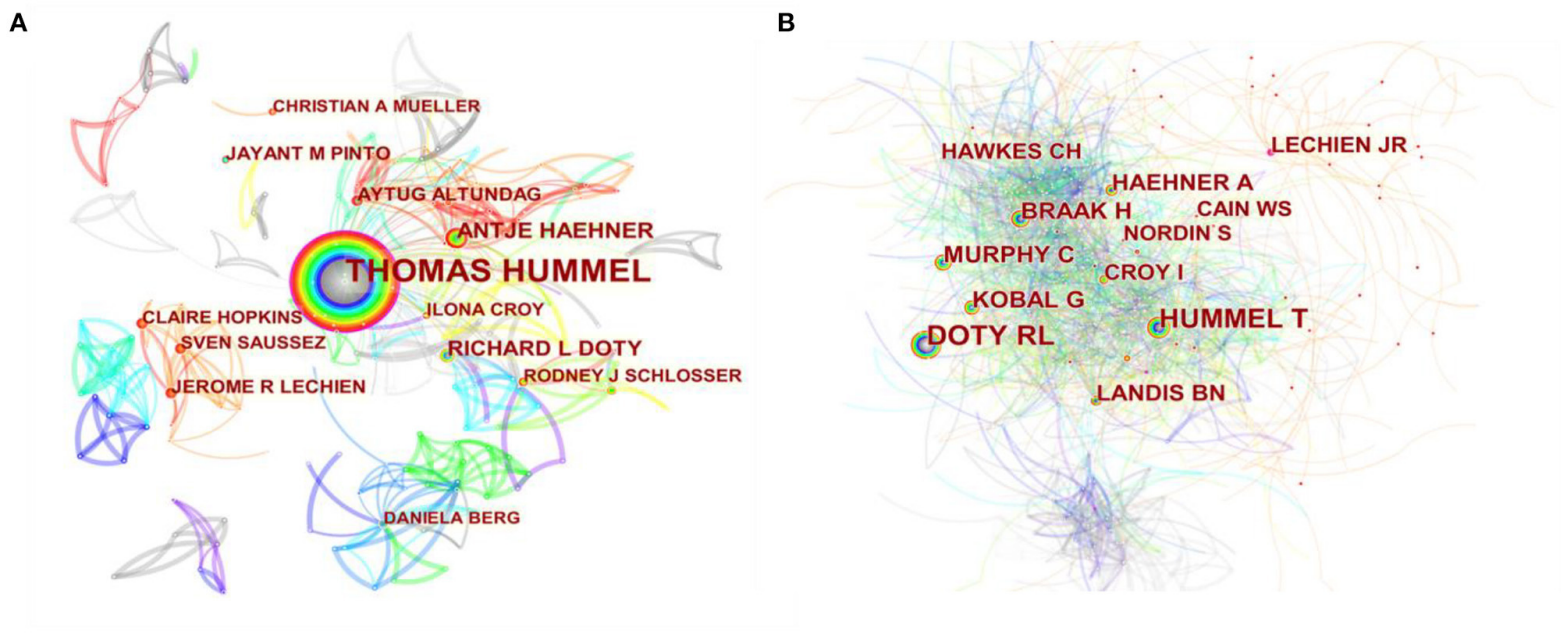
A network map of authors **(A)** or co-cited authors **(B)**.

In the past 20 years, a total of 19,056 authors have participated in publishing articles related to olfactory dysfunction. The top 10 most active authors are listed in Table 3. In the author collaboration network map (Figure 4A), the size of the circle reflects the number of articles published by authors, and the lines connecting the circles represent co-occurrence relationships between authors. Betweenness centrality is utilized to measure the likelihood of any shortest path through a node in the network. In CiteSpace, nodes with high betweenness centrality were shown as purple rings. The thickness of each purple ring describes the magnitude of the betweenness center value. Thomas Hummel from Germany, with a total of 270 publications, was comfortably ahead of all others in terms of authorship, followed by Antje Haehner (*n* = 45) also from Germany, Richard L. Doty (*n* = 39) from the U.S., Aytug Altundag (*n* = 23) from Turkey, Jayant M. Pinto (*n* = 21) from the U.S., and Jerome R. Lechien (*n* = 21) from France. Thomas Hummel, whose value of centrality was 0.14, occupied an absolute central position, indicating that he has cooperated more with other scholars than any other individual has (Figure 4B).

**Table 3:** Top 10 most active authors in the field of olfactory dysfunction from 2002 to 2021.

**No**.	**Author**	**Counts**	**Centrality**
1	Thomas Hummel	270	0.14
2	Antje Haehner	45	0
3	Richard L. Doty	39	0.06
4	Aytug Altundag	23	0.02
5	Jayant M. Pinto	21	0
6	Jerome R. Lechien	21	0
7	Claire Hopkins	20	0
8	Sven Saussez	19	0
9	Ilona Croy	19	0
10	Rodney J. Schlosser	18	0

### Journals

**Figure 5:**
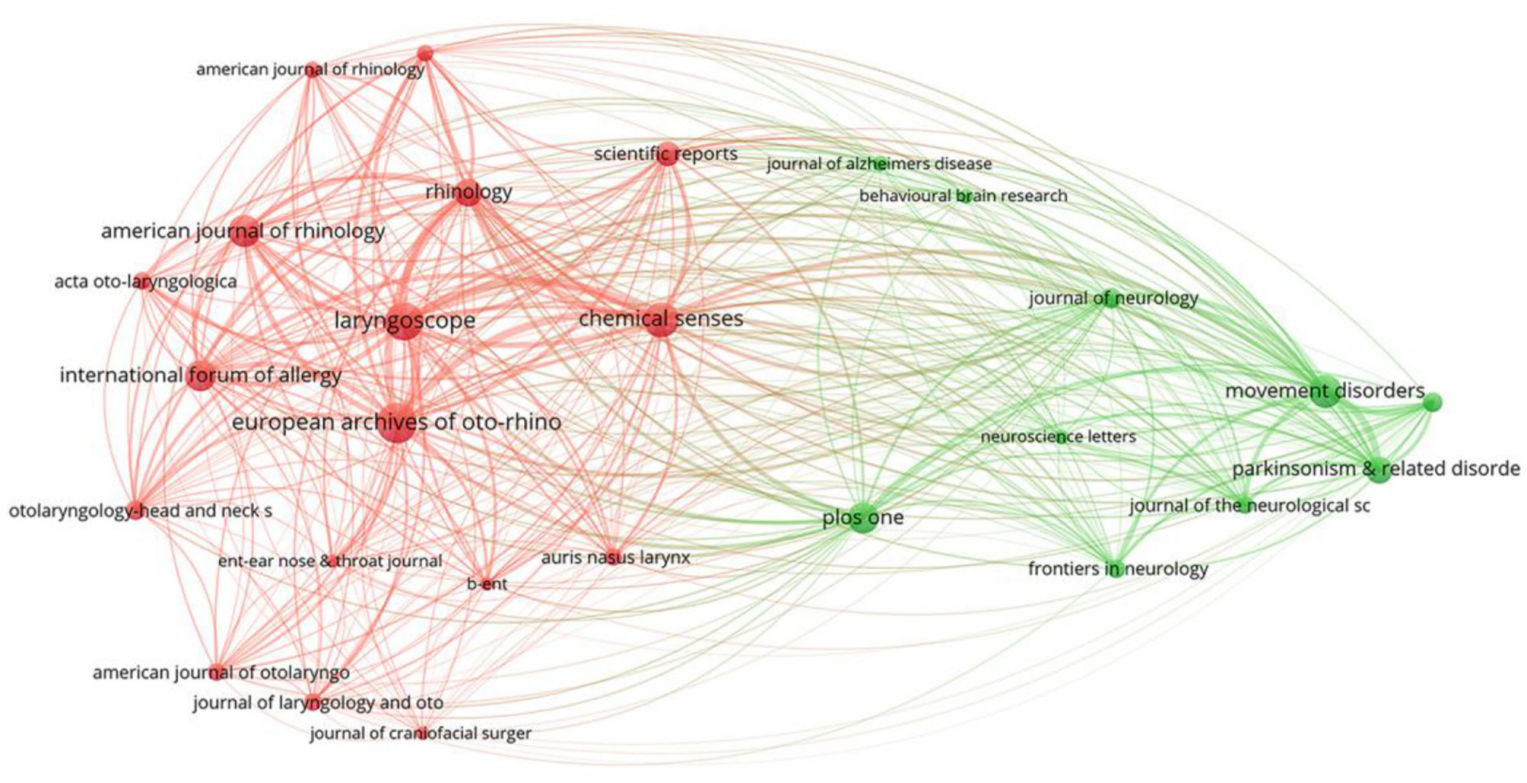
A network map showing the journals that have published research on olfactory dysfunction.

A total of 982 academic journals have published articles on olfactory dysfunction. The *European Archives of Oto-Rhino-Laryngology, Laryngoscope*, and *Chemical Senses* were among the top three of the 10 most popular journals and the top 10 co-citations of journals (Table 4). *Movement Disorder* had the highest impact factor among the top 10 most popular journals. Among the top 10 co-citations of journals, *Nature* had the highest impact factor. There were active mutual citation relationships between different journals (Figure 5). As shown in Figure 5, the journals that published articles related to olfactory dysfunctions can be clearly clustered into two categories: otolaryngology journals and the research direction of neuroscience.

**Table 4:** Top 10 most popular journals and co-citations of journals in the field of olfactory dysfunction from 2002 to 2021.

**No**.	**Journal**	**Articles counts**	**Impact factor (2021)**	**No**.	**Journal**	**Cited frequency**	**Impact Factor (2021)**
1	European Archives of Oto-Rhino-Laryngology	120	2.503	1	Laryngoscope	1,508	3.325
2	Laryngoscope	111	3.325	2	Chemical Senses	1,474	3.16
3	Chemical Senses	102	3.16	3	European Archives of Oto-Rhino-Laryngology	1,146	2.503
4	Movement Disorder	90	10.338	4	Neurology	1,145	9.910
5	American Journal of Rhinology and Allergy	84	2.467	5	PLoS ONE	888	3.24
6	PLoS ONE	80	3.24	6	Archives of Otolaryngology Head and Neck Surgery	862	2.327
7	International Forum of Allergy and Rhinology	79	3.858	7	Journal of Neurology Neurosurgery and Psychiatry	839	10.154
8	Rhinology	70	3.681	8	Movement Disorder	822	10.338
9	Parkinsonism and Related Disorders	67	4.891	9	Rhinology	822	3.681
10	Scientific Reports	57	4.379	10	Nature	817	49.962

### Analysis of Co-cited References

Table 5 demonstrated the top 10 co-cited references. Among these articles, “Lechien et al. (2020), *European Archives of Oto-Rhino-Laryngology*,” “Pollán et al. (2020), *Lancet*,” and “Gandhi et al. (2020), *New England Journal of Medicine*” were recent examples of highly citated articles, all three of which cover coronavirus disease 2019 (COVID-19). The studies published on the journals with impact factors of >20 are about COVID-19, animal models, and olfactory receptor genes, respectively. Among them, the aforementioned paper published by Gandhi et al., titled “Mild or Moderate COVID-19,” had the highest impact factor (91.253) and was published in the *New England Journal of Medicine*.

**Table 5:** Top 10 co-cited references in the field of olfactory dysfunction from 2002 to 2021.

**Rank**	**Article title**	**Author/Published year**	**Journal**	**Count**	**Impact factor (2021)**
1	Hypogonadotropic hypogonadism due to loss of function of the KiSS1-derived peptide receptor GPR54	de Roux et al., 2003	Proceedings of The National Academy of Sciences of The United States of America	1,633	11.205
2	Olfactory and gustatory dysfunctions as a clinical presentation of mild-to-moderate forms of the coronavirus disease (COVID-19): a multicenter European study	Lechien et al., 2020	European Archives of Oto-Rhino-Laryngology	1,197	2.503
3	Normative data for the “sniffin' sticks” including tests of odor identification, odor discrimination, and olfactory thresholds: an upgrade based on a group of more than 3,000 subjects	Hummel et al., 2007	European Archives of Oto-Rhino-Laryngology	1,029	2.503
4	Prevalence of SARS-CoV-2 in Spain (ENE-COVID): a nationwide, population-based seroepidemiological study	Pollán et al., 2020	Lancet	813	79.323
5	The olfactory receptor gene superfamily of the mouse	Zhang and Firestein, 2002	Nature Neuroscience	611	24.884
6	Where does Parkinson's disease pathology begin in the brain?	Del Tredici et al., 2002	Journal of Neuropathology and Experimental Neurology	517	3.685
7	Idiopathic hyposmia as a preclinical sign of Parkinson's disease	Ponsen et al., 2004	Annals of Neurology	511	10.422
8	The REM sleep behavior disorder screening questionnaire—a new diagnostic instrument	Stiasny-Kolster et al., 2007	Movement Disorders	492	10.338
9	Telomerase reactivation reverses tissue degeneration in aged telomerase-deficient mice	Jaskelioff et al., 2011	Nature	487	49.962
10	Mild or Moderate COVID-19	Gandhi et al., 2020	New England Journal of Medicine	481	91.253

**Figure 6:**
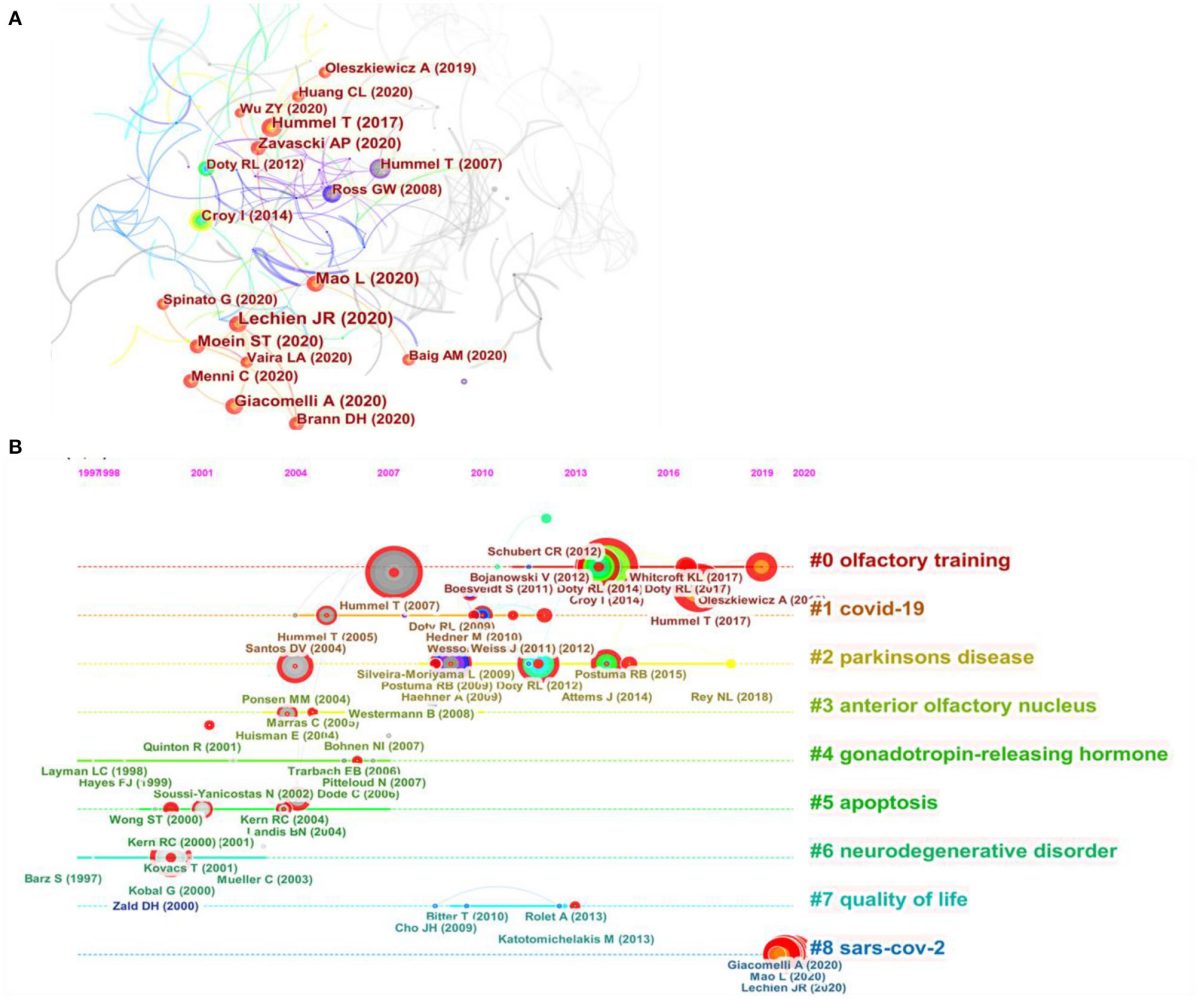
A network map showing the co-cited references **(A)** and the timeline view of co-cited clusters with cluster labels **(B)**.

Co-cited references were analyzed by CiteSpace for visual correlation analysis, from which the cluster network graph and the timeline view of co-cited references were obtained. In the cluster network graph (Figure 6A), each node represents a cited paper, and the red circles indicate a surge in citations. The timeline perspective of the clustering diagram, combined with an analysis of clustering and time, displayed the distribution of topics in the field and the trends and the inter-relationships of research topics over time (Figure 6B). The cluster theme is located in the rightmost diagram. The cluster analysis revealed nine clusters, consisting of olfactory training, COVID-19, Parkinson's disease, anterior olfactory nucleus, gonadotropin-releasing hormone, apoptosis, neurodegenerative disorder, quality of life, and SARS-CoV-2. The closest clusters on the timeline were “#0 olfactory training,” “#1 COVID-19,” “#2 Parkinson's disease,” “#7 quality of life,” and “#8 SARS-CoV-2”.

### Keywords

**Figure 7:**
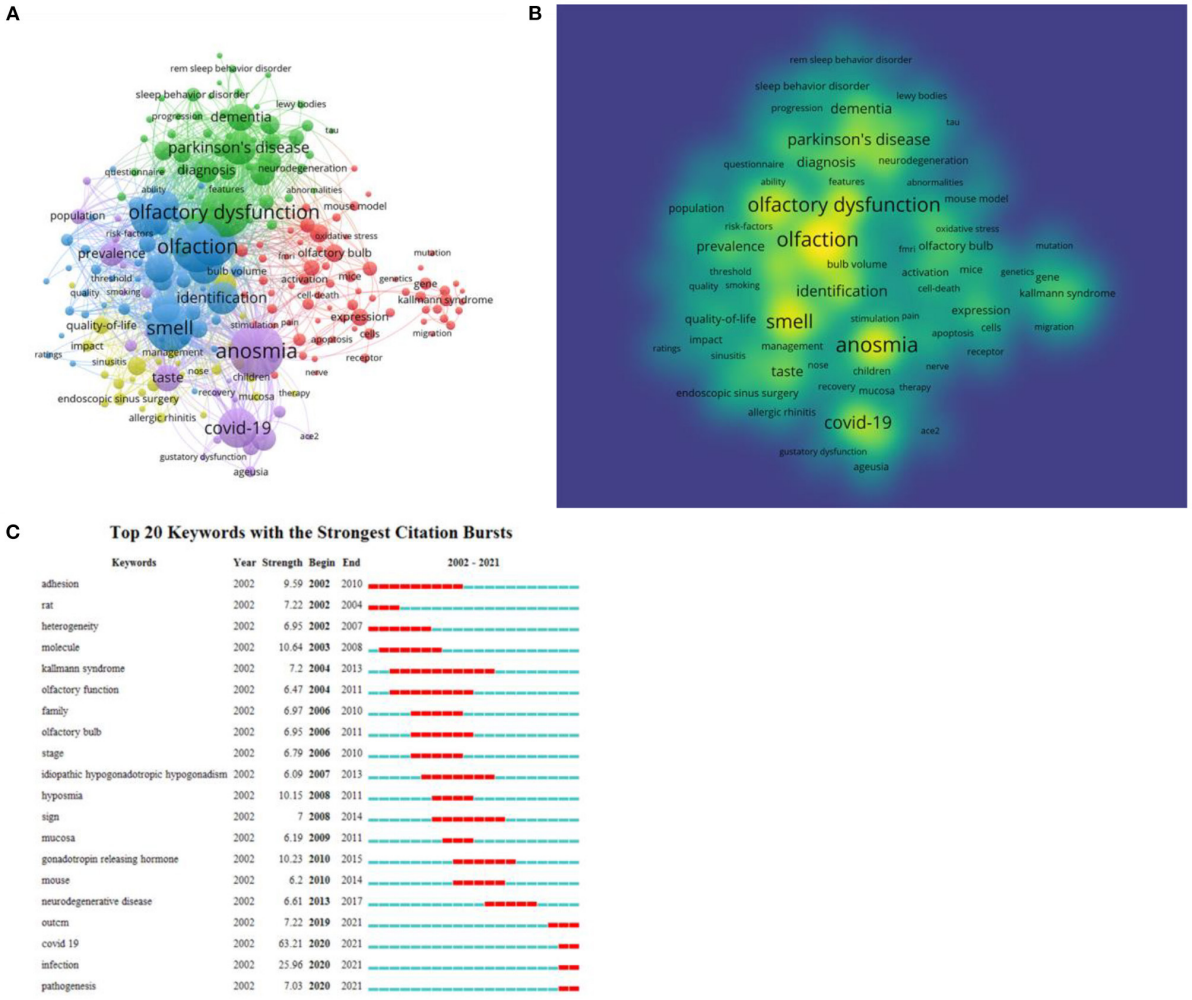
Co-occurrence analysis of global research on olfactory dysfunction published from 2002 to 2021. **(A)** Mapping of keywords in the research field. **(B)** Distribution of keywords according to the average frequency of appearance. **(C)** Keywords with the strongest citation bursts.

The keywords that occurred >20 times among 9,260 total keywords were analyzed using VOSviewer (Figure 7A). These keywords were divided into five clusters covering olfaction and diseases associated with olfactory dysfunction—namely neurodegenerative diseases, systemic diseases, nasal and nasocranial base diseases, and COVID-19. VOSviewer was also used to determine the density based on the frequency of the keywords (Figure 7B). Regions with higher optical density values in the density visualization suggested a research hotspot in this research area.

Burst keywords between 2002 and 2021 were identified by CiteSpace. In Figure 7C, the green line represents the time period from 2001 to 2021, and the interval of the burst keyword is shown by the red line. The keyword with the greatest burst strength in the past 20 years was “COVID-19” (63.21). Other burst keywords used in the past 5 years included “neurodegeneration disease” (2013–2017), “outcome” (2019–2021), “COVID-19” (2020–2021), “infection” (2020–2021), and “pathogenesis” (2020–2021).

## Discussion

Olfactory dysfunction is characterized by abnormalities in odor perception caused by organic or functional lesions in various parts of the olfactory pathway in the process of odor perception, conduction, and information analysis. Multiple studies have found that the prevalence of olfactory dysfunction detected by olfactory psychophysical tests is much higher than the self-reported prevalence rate (Landis et al., 2004; Nordin et al., 2004). In recent years, more and more researchers have become involved in the field, as olfactory dysfunction plays an important role in neurodegenerative diseases, psychiatric disorders, and infectious diseases alike. In this study, significant evolution between 2002 and 2021 was revealed by discovering articles published in the last two decades about olfactory dysfunction in the WoSSC database. After excluding studies that did not meet the screening criteria, 3,887 articles published in English remained. The information of these articles suggests the foci and new research directions in studying olfactory dysfunction.

Over the past 20 years, the overall volume of annually published research papers related to olfactory dysfunction has gradually increased. The year 2020 was a turning point because a drastic surge was witnessed in research on the topic in part due to the COVID-19 pandemic. The incremental increase in research volume can be ascribed to the fact that olfactory dysfunction is one of the symptoms of COVID-19 (Agyeman et al., 2020). However, excluding the factor of COVID-19, there remained a steady and gradual increase in the annual publication volume between 2002 and 2019, indicating that this topic has received significant attention in recent years.

The U.S. and Germany, the top two most productive countries, presented the greatest number of papers with an edge of foreign cooperation. China, Italy, and the U.K. have also published many papers, but their rate of international cooperation is significantly less than that of the U.S. and Germany. It seems that mutual collaboration among countries is proportional to the number of published articles. However, few countries in Africa have published research on olfactory disorders, and this has a certain relationship with the local economic environment. The good news, however, is that the annual increase in the number of countries involved in olfactory dysfunction research has kept pace with the number of articles being published each year, indicating that more and more researchers from different regions are engaging in studying anosmia. This trend partly reflects the increased focus of researchers on smell disorders. It is hoped that more countries will strengthen their international cooperation to support research progress in olfactory dysfunction. Interestingly, the U.S. partnered with twice as many countries publishing articles on olfactory disorders as Germany, but, in terms of institutions, the situation was reversed, with Dresden University in Germany publishing twice as many papers as the University of Pennsylvania in the U.S. This shows that there are more institutions involved in olfactory disorder–related research in the U.S., but these research institutions are relatively scattered, while the research institutions in Germany are relatively concentrated. Dresden University of Technology have a close collaboration with other Universities, such as the University of Pennsylvania (Pellegrino et al., 2021), Capital Medical University (Zhang et al., 2019) and University of Cagliari (Masala et al., 2018; Cecchini et al., 2019; Haehner et al., 2019; Solla et al., 2020). Institutions show greater cooperation than countries This further suggests that international cooperation needs to be strengthened.

Among the top 10 academic journals, the *European Archives of Oto-Rhino-Laryngology* ranked first in terms of total publications (120), followed by *Laryngoscope* (111) and *Chemical Senses* (102). These three journals also ranked as the top three among the top 10 co-citations of journals. Half of the top 10 academic journals were classified as otolaryngology journals, and the rest were classified as interdisciplinary journals covering olfactory disorders. These results suggest that researchers may select interdisciplinary journals when submitting manuscripts related to olfactory disorders so as to increase the attention of other disciplines on olfactory disorders and further enhance the research on olfactory disorders.

Thomas Hummel published the most papers in the domain of olfactory dysfunction over the last 2 decades. According to our analysis, the top five most productive authors hailed from Germany, the U.S., and Turkey. Thomas Hummel and Antje Haehner come from the same institution and have a strong collaborative relationship. Among co-cited authors, Richard L. Doty (1,354 citations) ranked first, followed by Thomas Hummel (1,016 citations) and Heiko Braak (435 citations). After a comprehensive analysis of the data of authors and co-cited authors, it can be found that Thomas Hummel and Richard L. Doty appear in both indicators. These two authors are distinguished professors in the field of olfactory disorders, and they are the developers of the “Sniffin' stick” test (Hummel et al., 2007) and the “University of Pennsylvania Smell Identification Test,” (Doty et al., 1984) respectively, which are two of the leading methods for diagnosing olfactory dysfunction. They have contributed to advancing the global exchange and collaboration in research on olfactory dysfunction.

A total of top 10 co-cited references from 2002 to 2021 show that researchers are growing more focused in their research on disorders related to olfactory dysfunction. In addition, among these 10 publications, the article “Normative data for the ‘Sniffin' sticks' including tests of odor identification, odor discrimination, and olfactory thresholds: an upgrade based on a group of more than 3,000 subjects” published by Hummel et al. (2007) is an important guide, as it found a correlation between olfactory function and age and determined normative TDI (Threshold, Discrimination, and Identification) values for olfactory testing at different ages in 3,832 subjects using “Sniffin' sticks” tests. Among the research on treatments for olfactory disorders, olfactory training has been a hot topic. Olfactory training is currently an effective treatment supported by level 1A evidence (Patel, 2017). Some studies have confirmed that olfactory training can improve olfactory function after upper respiratory tract infection, traumatic injury, and neurodegenerative diseases (Konstantinidis et al., 2013; Oleszkiewicz et al., 2018). Olfactory training increases neural remodeling in the brain and olfactory bulb volume (Mahmut et al., 2020). However, the specific mechanism behind it requires further study. Olfactory dysfunction is an early biomarker of neurodegeneration and is prevalent in many neurodegenerative diseases, particularly Parkinson's disease and Alzheimer's disease (Zhao et al., 2020). In recent years, impaired olfactory recognition function has gained increasing research interest as a potential marker of neurodegenerative disease progression. This is consistent with our analysis of the co-cited literature. Olfactory dysfunction is one of the earliest non-motor features in Parkinson's patients and precedes the onset of motor symptoms by several years (Braak et al., 2003). The olfactory bulb is the first brain region to be affected, and this progresses to other regions, such as the anterior olfactory nucleus and the lower brainstem. However, it is unclear whether olfactory impairment correlates with the severity of disease.

Burst keywords are important indicators of emerging trends and research frontiers. Based on this, CiteSpace was used to identify burst keywords, and it seems that “COVID-19” has been the strongest burst keyword since 2021. Apparently, olfactory dysfunction is referred to as one of the most significant symptoms of SARS-CoV-2 infection because it is one of the symptoms that has a strong and consistent association with a positive COVID-19 test (Sudre, 2021). A large multi-center survey in the European region reported that 85% of patients with new coronary pneumonia are plagued by anosmia (Lechien et al., 2020). In most cases, the loss of smell caused by COVID-19 can last for several weeks, but 10% of infected people also experience long-term symptoms, including long-lasting loss of smell or changes in smell (Tong et al., 2020; Boscolo-Rizzo et al., 2021). Therefore, the mechanism of olfactory dysfunction in patients with COVID-19 has become a research hotspot. Chen et al. (2020) was the first research team to discover that the SARS-CoV-2 receptor angiotensin-converting enzyme 2 (ACE2) is differentially expressed in the olfactory epithelium and respiratory epithelium. ACE2 is mainly expressed in the apical Krt18 sustentacular cells of the olfactory epithelium rather than in olfactory sensory neurons. According to this finding, the researchers speculated that SARS-CoV-2 affects olfactory function by disrupting gene expression. Zazhytska et al. (2022) revealed that the expression of genes related to olfactory receptors in the olfactory epithelium of SARS-CoV-2–infected animals and humans is chronically disturbed, mainly due to disturbances in the olfactory sensory neuron nuclear architecture. However, the pathogenesis of olfactory dysfunction in COVID-19 may be multifactorial and heterogeneous among patients. Therefore, further exploration of the signaling molecules leading to reorganization of the olfactory sensory neuron nuclear architecture will be helpful to understand the pathological mechanism of olfactory dysfunction and explain the reasons for the long-term sequelae in some patients.

### Limitation

The present bibliometric analysis offers a better understanding of the evolving research hotspots and trends in olfactory dysfunction research by systematically analyzing a large number of publications in an objective and visual way. However, it still has some limitations. First, the data used in our research were retrieved only from the WoSCC database, and studies in other important literature databases were ignored. Second, the articles retrieved were limited to those published in English, leading to some bias in the analysis. In addition, due to the insufficient data available for 2022, articles published in 2022 were excluded. However, this study included most papers related to olfactory dysfunction over the past 20 years, so data from 2022 would likely have little impact on the overall analysis results. Finally, as the limitations of the CiteSpace and VOSviewer, some information unable to display, such as the most representative references for each country.

## Conclusion

In the past two decades, researchers have grown increasingly interested in the field of olfactory dysfunction. The quantity of publications related to olfactory dysfunction is increasing rapidly, but cooperation among countries, especially developing countries, needs to be strengthened. As an early symptom of neurodegeneration and COVID-19, olfactory dysfunction has always been a research hotspot. However, the pathological mechanism of olfactory dysfunction still needs to be further explored by researchers. In the treatment of olfactory dysfunction, olfactory training is an effective treatment, but the mechanism of how it improves olfactory function is still unknown. These will be the future directions of olfactory dysfunction research.

## Data Availability Statement

The original contributions presented in the study are included in the article, further inquiries can be directed to the corresponding authors.

## Author Contributions

WH and YW designed the study. WY and PP performed the search and collected data. XZ re-checked the data. WH and NC analyzed the data. WH wrote the article. YW and XZ revised the article. All authors contributed to the article and approved the submitted manuscript.

## Funding

This study was supported by Public service development and reform pilot project of Beijing Medical Research Institute (BMR2021-3), the Key Special Projects for International Cooperation in Science and Technology Innovation between the Ministry of Science and Technology (No. 2019YFE0116000), and Key Program of National Nature Science Foundation of China (No. 82130031).

## Conflict of Interest

The authors declare that the research was conducted in the absence of any commercial or financial relationships that could be construed as a potential conflict of interest.

## Publisher's Note

All claims expressed in this article are solely those of the authors and do not necessarily represent those of their affiliated organizations, or those of the publisher, the editors and the reviewers. Any product that may be evaluated in this article, or claim that may be made by its manufacturer, is not guaranteed or endorsed by the publisher.
